# Climate of Tropical Florida

**Published:** 1840-01

**Authors:** 


### CLIMATE OF TROPICAL FLORIDA.

Abstract from the Metereological Register kept at Indian Key, T. F., by Charles Howe, Esq., P. M., and Inspector of the Port. Thermometrical observations made at sunrise and 2 P. M. each day.

Explanation of the *initials* and *signs* at the *heads* of the *columns*. *c. d.* The coldest day at sunrise. *h. d.* The hottest day at 2 P. M. *v. m.* The greatest variation in one month. *v. d.* The greatest variation in one day. v. d. The least variation in one day. *r. d.* Rainy days or number of days in which showers fell. *m. i. r.* Monthly inches of rain or the number of inches of water thrown by the Pluviometer at the end of each month.

| 1836. | *cd* | *hd* | *vm* | *vd* | vd | *rd* | *mir* | 1838. | *cd* | *hd* | *vm* | *vd* | vd | *rd* | *mir* |
|---|---|---|---|---|---|---|---|---|---|---|---|---|---|---|---|
| January, | 54 | 77 | 23 | 9 | 0 | 1 | 2.00 | January, | 64 | 82 | 18 | 7 | 1 | 1 | 0.20 |
| February, | 50 | 81 | 31 | 11 | 0 | 1 | 0.64 | February, | 50 | 83 | 33 | 14 | 2 | 2 | 0.06 |
| March, | 60 | 80 | 20 | 9 | 0 | 3 | 2.50 | March, | 62 | 82 | 20 | 10 | 1 | 1 | 0.30 |
| April, | 71 | 83 | 12 | 5 | 0 | 4 | 2.00 | April, | 64 | 84 | 20 | 11 | 2 | 1 | 1.08 |
| May, | 75 | 84 | 9 | 6 | 0 | 5 | 6.50 | May, | 70 | 87 | 17 | 10 | 2 | 3 | 2·40 |
| June, | 78 | 88 | 10 | 8 | 0 | 4 | 3.50 | June, | 76 | 88 | 12 | 8 | 0 | 6 | 7.00 |
| July, | 77 | 86 | 9 | 4 | 0 | 3 | 5.34 | July, | 80 | 90 | 10 | 7 | 2 | 1 | 2.40 |
| August, | 80 | 86 | 6 | 6 | 0 | 4 | 1.69 | August, | 82 | 90 | 8 | 6 | 2 | 2 | 3.00 |
| September. | 80 | 86 | 6 | 4 | 0 | 13 | 5.90 | September, | 78 | 89 | 11 | 6 | 1 | 8 | 7.10 |
| October, | 74 | 85 | 11 | 4 | 0 | 2 | 3.86 | October, | 68 | 86 | 18 | 10 | 0 | 5 | 6.22 |
| November, | 62 | 80 | 18 | 7 | 0 | 1 | 1.22 | November, | 67 | 84 | 17 | 8 | 0 | 3 | 7.10 |
| December, | 60 | 78 | 18 | 10 | 0 | 0 | 0.00 | December, | 63 | 84 | 21 | 12 | 0 | 3 | 2.60 |

| 1837. | *cd* | *hd* | *vm* | *vd* | vd | *rd* | *m ir* | 1839. | *cd* | *hd* | *vm* | *vd* | vd | *rd* | *mir* |
|---|---|---|---|---|---|---|---|---|---|---|---|---|---|---|---|
| January, | 50 | 84 | 34 | 11 | 2 | 6 | 3.72 | January, | 54 | 80 | 26 | 12 | 0 | 2 | 0.08 |
| February, | 51 | 80 | 29 | 10 | 1 | 3 | 1.10 | February, | 61 | 84 | 23 | 12 | 1 | 4 | 0.12 |
| March, | 59 | 82 | 23 | 10 | 1 | 1 | 0.09 | March, | 59 | 84 | 25 | 10 | 1 | 1 | 0.02 |
| April, | 65 | 86 | 21 | 9 | 2 | 3 | 2.00 | April, | 58 | 87 | 29 | 12 | 2 | 4 | 3.00 |
| May, | 72 | 88 | 16 | 10 | 0 | 3 | 0.90 | May, | 76 | 88 | 22 | 9 | 0 | 11 | 6.90 |
| June, | 76 | 90 | 14 | 9 | 2 | 2 | 3.10 | June, | 79 | 90 | 11 | 6 | 2 | 8 | 4.50 |
| July, | 79 | 90 | 11 | 7 | 0 | 4 | 4.70 | July, | 80 | 89 | 9 | 7 | 2 | 8 | 7.60 |
| August, | 78 | 90 | 12 | 6 | 2 | 5 | 6.75 | August, | 81 | 89 | 8 | 7 | 1 | 6 | 2.60 |
| September, | 79 | 88 | 9 | 6 | 1 | 3 | 7.25 | September, | 78 | 88 | 10 | 7 | 2 | 4 | 6.50 |
| October, | 61 | 88 | 27 | 6 | 1 | 5 | 8.75 | October, | | | | | | | |
| November, | 61 | 87 | 26 | 9 | 0 | 2 | 6.10 | November, | | | | | | | |
| December, | 62 | 84 | 22 | 9 | 2 | 3 | 2.12 | December, | | | | | | | |

GENERAL RESULTS.

| | 1836. | 1837. | 1838. | Oct. 1, 1839. |
|---|---|---|---|---|
| Number of clear days in the year | 289 | 290 | 314 | 210 |
| " cloudy " " | 35 | 35 | 15 | ·15 |
| " rainy " " | 41 | 40 | 36 | 48 |
| Extreme hottest day " " | 88° | 90° | 90° | |
| " coldest " " " | 50° | 50° | 50° | |
| Greatest change in one month, | 31° | 34° | 33° | |
| " " " " day, | 11° | 11° | 14° | |

Number of rainy days in *each rainy season*, from the 1st of June to the 1st of October, each year.

| | | | |
|---|---|---|---|
| 24 | 14 | 17 | 26 |

that is the number of days on which showers fell, because there is never an entirely rainy day; and because the showers mostly fall by night. N. B. In the first nine month of the present year it should be noted that the showers have been uncommonly numerous and have occurred at unusual times.

To Professor Daniel Drake.

Indian Key, Tropical Florida, 28th October, 1839.

Dear Sir:

I respectfully transmit to you the above abstract from the Meterological Register kept at this miniature Islet (of 12 acres) during the whole period of the Seminole war by Charles Howe, Esq. P. M. These indisputable evidences of the usual weather in Tropical Florida ought to demonstrate, that in South Florida, military operations against the savage Seminoles could have been continued throughout the whole summers, as well as at all other seasons of these years, from the 1st January, 1836, to 1st October, 1839. As a patriotic physcian it has become my indispensable duty—1st. To re-affirm boldly the positive fact, that below 26°, South Florida, with its *peculiarly tropical air*, irrefutably enjoys the *most healthy climate* in the U. States. 2d. To announce firmly the additional fact, that South Florida, with its *exclusively calcareous earth*, also possesses the *most healthy soil* in the United States; and 3dly, to invite earnestly my medical brethren, to *publish speedily* every fact and every argument which can or may be brought, or which may be thought, to show, that either in climate or in soil, South Florida is not the healthiest district in the Union.

Very respectfully, your ob't serv't,

HENRY PERRINE, M. D.

